# The histamine analogue clobenpropit modulates IRF7 phosphorylation and interferon production by targeting CXCR4 in systemic lupus erythematosus models

**DOI:** 10.3389/fimmu.2024.1490593

**Published:** 2024-12-16

**Authors:** Nassima Bekaddour, Nikaïa Smith, Birgit Caspar, Severine Grinberg, Stephane Giorgiutti, Vincent Rodeschini, Stephanie Dupuy, Nicolas Leboulanger, Darragh Duffy, Pauline Soulas-Sprauel, Vincent Gies, Anne-Sophie Korganow, Sébastien Nisole, Jean-Philippe Herbeuval

**Affiliations:** ^1^ Centre National de la Recherche Scientifique (CNRS) Unité Mixte de Recherche (UMR), Université Paris Cité, Paris, France; ^2^ Team Chemistry & Biology, Modeling & Immunology for Therapy (CBMIT), Paris, France; ^3^ Translational Immunology Unit, Institut Pasteur, Université Paris-Cité, Paris, France; ^4^ Université de Strasbourg, Institut National de la Santé et de la Recherche Médicale (INSERM), Unité Mixte de Recherche (UMR) S1109, Institut Thématique Interdisciplinaire (ITI) de Médecine de Précision de Strasbourg, Transplantation Excellence Nouvelle Génération (Transplantex NG), Fédération Hospitalo-Universitaire OMICs for Care (OMICARE), Fédération de Médecine Translationnelle de Strasbourg (FMTS), Strasbourg, France; ^5^ Department of Clinical Immunology and Internal Medicine, National Reference Center for Systemic Autoimmune Diseases (Centre de Référence des Maladies Auto-Immunes Rares de Strasbourg, (CNR RESO)), Tertiary Center for Primary Immunodeficiency, Strasbourg University Hospital, Strasbourg, France; ^6^ Université de Strasbourg, Faculty of Medicine, Strasbourg, France; ^7^ Edelris, Lyon, France; ^8^ Unité d’Appui et de Recherche (UAR) BioMedTech Facilities, Plateforme cyto2Bm, Université Paris Cité, Paris, France; ^9^ Faculté de Médecine, Université Paris Cité, Paris, France; ^10^ Department of Paediatric Otolaryngology, Assistance Publique – Hôpitaux de Paris (AP-HP), Hôpital Necker-Enfants Malades, Paris, France; ^11^ Université de Strasbourg, Faculty of Pharmacy, Strasbourg, France; ^12^ Institut de Recherche en Infectiologie de Montpellier (IRIM), Université de Montpellier, Centre National de la Recherche Scientifique (CNRS) Unité Mixte de Recherche (UMR) 9004, Montpellier, France

**Keywords:** interferons, pDc, SLE, CXCR4, pIRF7

## Abstract

**Introduction:**

Systemic lupus erythematosus (SLE) is an autoimmune disease characterized by an overactive immune response, particularly involving excessive production of type I interferons. This overproduction is driven by the phosphorylation of IRF7, a crucial factor in interferon gene activation. Current treatments for SLE are often not very effective and can have serious side effects.

**Methods:**

Our study introduces clobenpropit, a histamine analogue, as a potential new therapy targeting the CXCR4 receptor to reduce IRF7 phosphorylation and subsequent interferon production. We employed various laboratory techniques to investigate how clobenpropit interacts with CXCR4 and its effects on immune cells from healthy individuals and SLE patients.

**Results:**

Clobenpropit binds effectively to CXCR4, significantly inhibiting IRF7 phosphorylation and reducing interferon production. Additionally, clobenpropit lowered levels of pro-inflammatory cytokines in a mouse model of lupus, demonstrating efficacy comparable to the standard treatment, prednisolone.

**Discussion:**

These results suggest that clobenpropit could be a promising new treatment for SLE, offering a targeted approach with potential advantages over current therapies.

## Introduction

Systemic lupus erythematosus (SLE) is a complex autoimmune disease characterized by a variety of autoantibodies, complement activation and immune complex accumulation, resulting in tissue and organ damage, chronic inflammation and the production of autoantibodies against self-antigens (1). SLE affects multiple organs and systems in the body, including the skin, joints, kidneys, heart, and nervous system. The underlying pathology involves immune system dysregulation, leading to the formation of immune complexes that deposit in tissues, triggering inflammation and tissue damage. SLE is characterized by flares and remissions, with symptoms varying widely among individuals. The exact cause of SLE is unknown, but it is thought to involve a combination of genetic, environmental, and hormonal factors. The typical approach to treatment frequently involves the use of steroids either alone or in conjunction with hydroxychloroquine (HCQ) (2). Regrettably, these therapeutic measures may not consistently yield satisfactory results, and the prolonged use of steroids is associated with numerous side effects. Belimumab (anti-BAFF) demonstrated a reduction in disease activity related to SLE in 40% of the treated patients after one year (3). However, individuals with severe SLE, particularly those with renal or central nervous system involvement, are not always eligible for or do not respond to this treatment, necessitating the use of immunosuppressive drugs such as cyclophosphamide, azathioprine, or mycophenolate mofetil.

Type I interferons (IFN-I) significantly contribute to SLE pathology (4). The dysregulated production of IFN-I, particularly IFNα, a hallmark of SLE, drives the autoimmune process (5, 6). The excessive IFN-I signaling leads to the upregulation of various pro-inflammatory cytokines, chemokines, and immune cell activation markers, contributing to immune system dysregulation and the production of autoantibodies. Furthermore, prolonged IFN-I exposure enhances the activation and survival of autoreactive B cells (7) and promotes the differentiation of T cells towards pro-inflammatory subsets, perpetuating the autoimmune response (8, 9). Numerous studies showed that pDCs largely contribute to the ongoing production of IFN in SLE (5, 6, 10). Consequently, in murine models of lupus, reducing pDC levels improves the disease, and genetically compromised pDC function leads to disease improvement. A recent study also demonstrated that targeting pDCs in SLE patients diminishes the expression of IFN response genes in the blood, decreases immune cell infiltration in the skin, and alleviates skin lesions (11–13).

Clobenpropit (CB) is a stable analogue of histamine that has previously been shown to modulate immune responses through interactions with the CXCR4 receptor (14). This compound was chosen for its ability to affect IFN-I production, making it a potential therapeutic candidate for SLE. Additionally, R848 (resiquimod) is a synthetic agonist of TLR7/8, which simulates viral RNA by binding to TLR7, primarily in plasmacytoid dendritic cells (pDCs). Activation of TLR7 by R848 leads to downstream phosphorylation of IRF7, a critical regulator of type I interferon responses (15–17). The use of R848 in our experiments serves to mimic the overactive TLR7 signaling pathway seen in SLE, thereby allowing us to assess the potential of CB to mitigate these pathways.

Interferon-regulating factor 7 (IRF7) is the main transcription factor involved in the regulation of the IFN-I response in pDCs (18), thereby significantly influencing SLE pathogenesis (19, 20). IRF7 serves as the primary controller of IFN-I immune reactions, not only overseeing the continued expression of IFN-β but also prompting the production of IFN-α. Knocking out IRF7 specifically in mouse plasmacytoid dendritic cells (pDCs) nearly abolishes their ability to generate IFN-α (18, 21). Similarly, the absence of IRF7 in humans significantly hampers IFN-α production (18, 22, 23). Following viral infection and other stimuli, cytosolic IRF7 becomes activated through various types of innate pattern recognition receptors (PRRs). These PRRs, associated with IRF7, can be categorized into cytosolic and transmembrane signaling receptors. The phosphorylation of IRF7 primarily occurs in innate immune cells upon encountering virus-specific antigenic materials (DNA, RNA) via PRRs, followed by activation of intracellular signaling molecules. Dimerized IRF7 complexes can then migrate into the nucleus to initiate the expression of type I IFN genes. Activation of TLR-7 in pDCs triggers IRF7 phosphorylation (pIRF7), causing its nuclear translocation and initiating IFN-I gene transcription (18). Increased IRF7 expression in SLE patients contributes to the dysregulation of the immune system. The overproduction of IRF7-induced IFN-I triggers a cascade of immune responses, including the upregulation of pro-inflammatory cytokines and chemokines, activation of additional immune cells, and the production of autoantibodies against self-antigens (24). Hence, molecules capable of directly or indirectly regulating IRF7 expression present promising therapeutic avenue in SLE and more broadly in type I interferonopathies (22).

The chemokine receptor CXCR4, widely expressed by many cell types including metastatic cells, hematopoietic progenitors and all immune cell subtypes such as pDCs and monocytes, is a member of the G protein-coupled receptor (GPCR) family, with CXCL12 as its natural ligand (25–27). The CXCR4/CXCL12 axis plays a key role in multiple biological processes including embryogenesis (28), homing and immune cell chemotaxis (29). Upregulation of CXCR4 expression and dysregulation of CXCR4/CXCL12 axis have been described in autoimmune diseases in particular in SLE (30). Thus, CXCR4 represents a putative drug target (31), evidenced by the development of numerous drugs aimed at modulating this receptor (32, 33). However, clinical trials investigating CXCR4 antagonists have sometimes been halted or encountered challenges due to adverse effects associated with disrupting the CXCR4/CXCL12 axis. These include immunosuppression, impaired wound healing, and cardiovascular complications (34–36).Although the up regulation of IFN signaling in SLE was identified decades ago, the progress of anti-IFN therapies has been slow. This delay is attributed to the heterogeneity observed in the disease’s progression, preventing these therapies from becoming a routine part of clinical practice. Histamine, an endogenous monoamine, plays a role in the immune system and is involved in inflammatory responses (37–39). While the link between histamine and SLE is not directly established, some studies suggest immunomodulatory effects of histamine and histamine analogues (40, 41). We previously demonstrated that histamine and the histamine analogue CB could regulate IFN-I production by primary pDCs exposed to Influenza A virus (IAV) *in vitro* and *in vivo (* 14). We also demonstrated that the modulatory activity of CB is linked to the chemokine receptor CXCR4, as the suppression of CXCR4 expression on primary pDCs using CXCR4 siRNA reduced CB’s ability to prevent IFN activity (14).

The pristane-induced lupus mouse model is widely used to study SLE as it closely recapitulates many features of human disease and type I interferon signatures (42). In this model, the injection of pristane, an isoprenoid alkane, leads to chronic inflammation and autoimmunity. While not an exact match to all aspects of human SLE, this model has proven valuable for preclinical studies of new therapies due to its reproducible immune phenotypes and the ability to induce disease in a relatively short time frame.

In this study, we hypothesize that the histamine analogue CB can modulate immune responses in SLE by targeting the CXCR4 receptor leading to reduction of IRF7 phosphorylation and the subsequent production of interferons. Our objectives were to investigate the effects of CB on IFN-I production, particularly in the context of SLE, where overactivation of the TLR7 pathway in pDCs drives excessive production of type I interferons, contributing to disease pathology. By targeting this pathway, CB could potentially mitigate key inflammatory processes in SLE. To build on our earlier findings, this study extends the investigation of CB to include cells directly derived from SLE patients, providing more relevant insights into its potential therapeutic effects in autoimmune contexts. In particular, we aimed to evaluate CB’s capacity to interact with CXCR4 and to prevent IRF7 phosphorylation, which is a key step in the activation of the IFN-I pathway. Using both *in vitro* models (PBMCs, pDCs), ex vivo samples from SLE patients’ bone marrow and peripheral blood, and an *in vivo* pristane-induced SLE mouse model, we sought to elucidate whether CB could serve as a potential therapeutic strategy for SLE by modulating CXCR4-dependent pathways.

## Results

### CB reduces interferon production in tonsillar monuclear cells and whole blood

**Figure 1:**
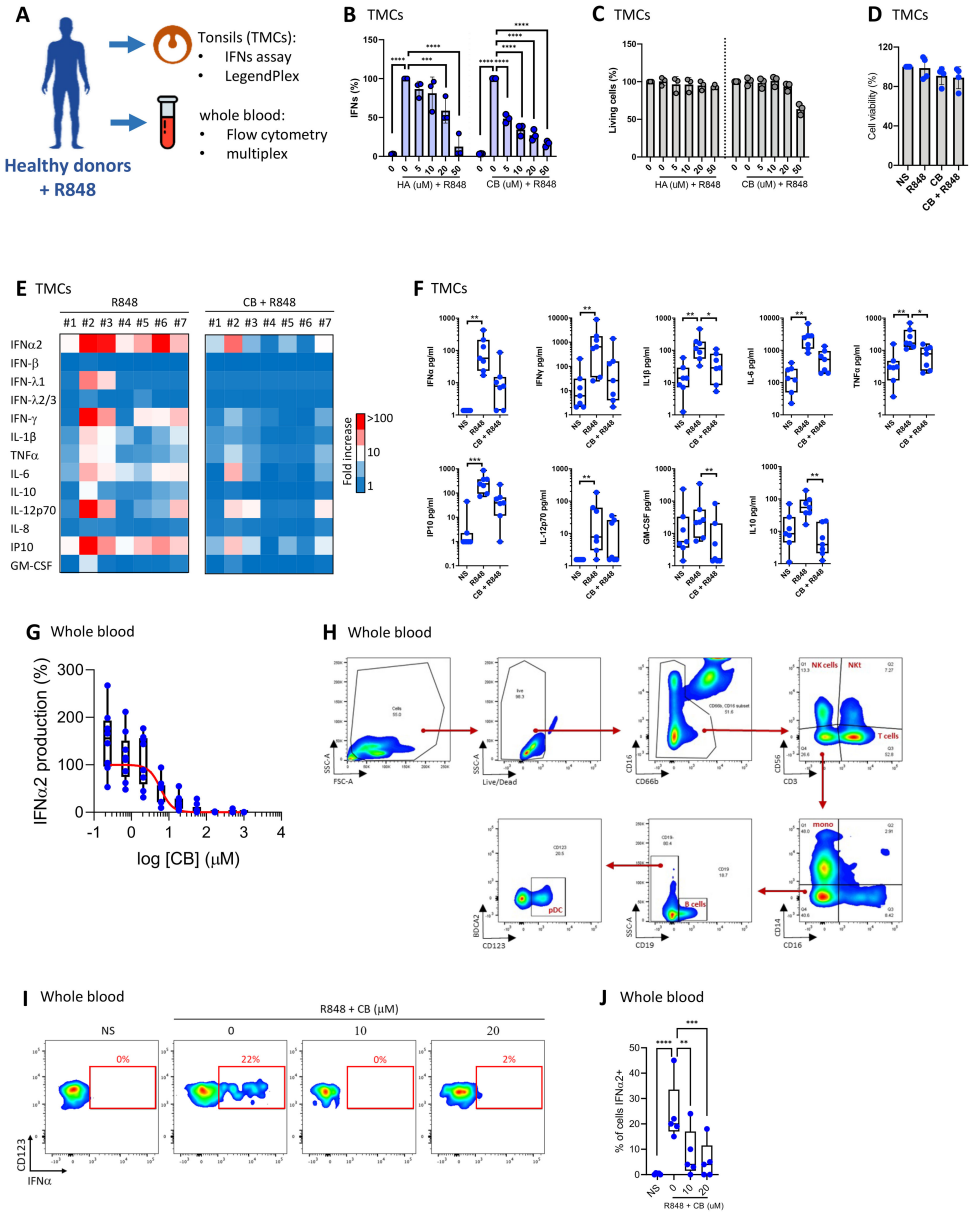
CB suppresses TLR7-mediated inflammation response in tonsils and whole blood. **(A)** Overview of key tests conducted. **(B, C)** Effect of HA and CB on TMCs from three healthy individuals was evaluated. Cells were pre-treated with either HA or CB for 1 hour, followed by stimulation with R848. IFNs in the supernatant were measured using the reporter cell line twINNEE. **(C)** Cell viability was measured using an ATP quantification assay. Two-way ANOVA with dunnett’s multiple comparisons test. **(D–F)** Effect of CB on TMCs from seven healthy individuals was evaluated. Cells were pre-treated with CB for 1 hour, followed by stimulation with R848. Cytokine levels in the cell supernatant were measured using a LEGENDplex assay. **(D)** Cell viability measured using an ATP quantification assay. **(E)** Heat map represents the fold increase in cytokine secretion compared to NS. **(F)** Graphs represent the concentration in picograms per milliliter of each measured cytokine. Box and whisker plots with median ± minimum to maximum. Friedman test with Dunn’s *post hoc* correction. Friedman test with dunnett’s multiple comparisons test. **(G–J)** Effect of CB on whole blood samples from seven healthy individuals was evaluated. Whole blood was pre-treated with CB for 1 hour, followed by stimulation with R848. **(G)** Graphs represent the concentration in picograms per milliliter of IFN-α2 in the supernatant of seven healthy individuals using SIMOA. **(H, I)** IFN-α production in blood pDCs quantified using flow cytometry with **(H)** the gating strategy shown, **(I)** dotplot representation of one representative donor and **(J)** graphical representation of the five donors showing the percentage of IFN-α-producing cells. Two-way ANOVA with dunnett’s multiple comparisons test. All data are presented as median ± range. ****P < 0.0001, ***P < 0.001, **P < 0.01, *P < 0.05.

Our prior study revealed that CB modulates IFN-α production by virus-activated pDC (14) and inflammatory factor release from monocytes (43). Given the importance of tonsillar tissue in immunological responses, and its role as the primary site of pathogen exposure and immune activation, we next tested CB activity in a tonsillar mononuclear cells (TMCs) model. The experimental setup and key tests conducted are summarized in a schematic (**Figure 1A**). We exposed TMCs from seven healthy donors to increasing concentrations of histamine (HA) or CB, followed by stimulation with R848. Both HA and CB prevented interferon (IFN) production, with CB being more potent than HA (**Figure 1B**). Importantly, HA or CB treatments did not significantly affect cell viability, except for TMCs treated with the highest CB concentration (50 µM) (**Figure 1C**). CB significantly reduced the production of several inflammatory cytokines, including IFN-α2, IFN-β, IFNλ1, IFN-λ2/3, and IFN-γ (**Figure 1D**) without toxicity (**Figures 1E, F**). To investigate CB’s impact on interferons whole blood samples from 7 healthy donors were treated with a range of CB doses followed by R848 stimulation. The levels of IFN-α within culture supernatants were evaluated using Single Molecule Array (SIMOA) (**Figure 1G**). This analysis revealed CB’s potent prevention of IFN-α production with an IC_50_ of 6 µM. To this end, pDCs were selected from whole blood in flow cytometry through marking cell specific proteins with fluorophore coupled antibodies followed by the displayed gating strategy (**Figure 1H**). Subsequently, IFN-α production was assessed in R848 pre-treated pDCs upon exposure to CB (**Figures 1I, J**). This approach distinctly demonstrated CB’s capacity to prevent IFN-α production within the selected pDCs sourced from whole blood of healthy donors.

### CB reduces interferon production in PBMCs and primary pDCs

**Figure 2:**
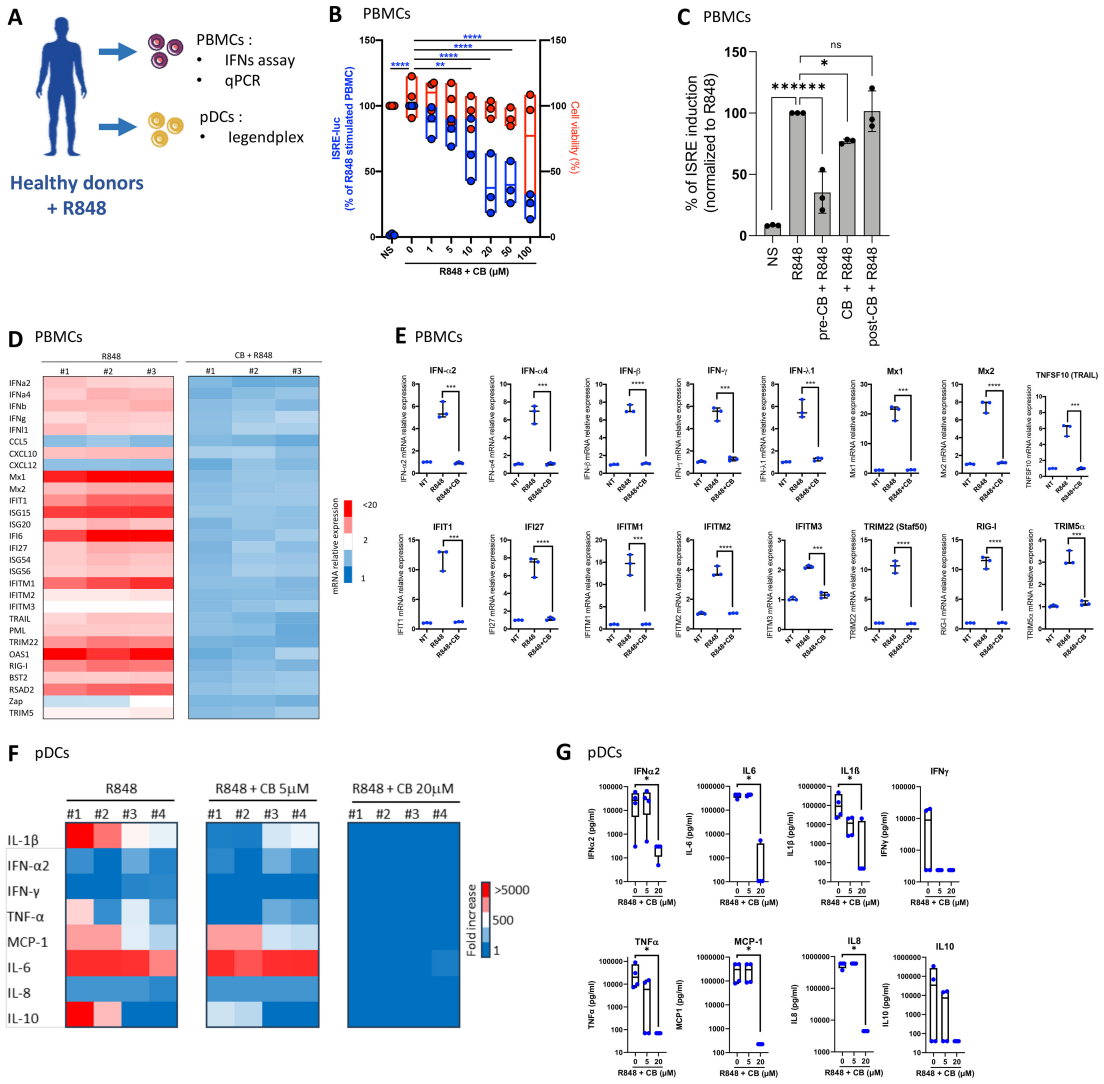
CB Modulates TLR7-Mediated Inflammation in PBMCs and pDCs. **(A)** Overview of key tests conducted. **(B–E)** The impact of CB on R848-stimulated PBMCs was assessed in the supernatant of three healthy individuals. IFNs in the culture supernatant were quantified using the twINNEE reporter cell line. **(B)** Graphical representations of three healthy donors are shown. Two-way ANOVA with dunnett’s multiple comparisons test. **(C)** Human PBMCs (n=3) were treated with 20 µM CB either 1 h before stimulation (pre-CB + R848), simultaneously (CB + R848), or 1 h after stimulation with R848 (post-CB + R848) for 24 (h) Culture supernatants were collected and IFN levels were measured using the twINNEE cells by activation of the ISRE (interferon-stimulated response element) pathway. Histograms represent the relative levels of ISRE induction for each treatment condition, illustrating the temporal modulation of the CB effect on R848-induced IFN pathway activation. **(D, E)** The influence of CB on R848-stimulated PBMCs was determined via qPCR. **(D)** The heat map illustrates the mRNA expression levels of each inflammatory factor. **(E)** Graphs depict the levels of each measured cytokine. Two-way ANOVA with Dunn’s *post hoc* correction was performed. **(F–G)** The effect of CB on R848-stimulated pDCs was evaluated in supernatant using Legendplex. **(F)** The heat map displays the fold increase in cytokine secretion compared to the negative control NS. **(G)** Graphs represent the concentration in picograms per milliliter of each measured cytokine. Kruskal-Wallis test with Dunnett’s multiple comparisons test. All data are presented as median ± range. ****P < 0.0001, ***P < 0.001, **P < 0.01, *P < 0.05.

To investigate the effects of CB on the interferon production, peripheral blood mononuclear cells (PBMCs) from three healthy donors were exposed to increasing concentrations of CB, followed by stimulation with R848. The experimental setup and key tests conducted are summarized in a schematic (**Figure 2A**). The subsequent production of interferons in the supernatants was quantified using a luminescence-based assay, employing the twINNE reporter cell line (44). This cell line, engineered to express the interferon-stimulated response element (ISRE) linked to luciferase, facilitates the direct measurement of interferon activity (**Figure 2B**). CB treatment showed negligible effects on PBMC viability. However, it exhibited a potent prevention of R848-mediated interferon production. We then investigated the effects of CB on PBMCs when incubated either simultaneously with R848 or after the stimulation (1h). This approach allowed us to assess how the timing of CB administration influences its effectiveness in modulating the immune response triggered by R848. We observed that when PBMCs were exposed to CB and R848 simultaneously, CB reduced ISRE stimulation, albeit less effectively than with a 1-hour pre-incubation. In contrast, when cells were first activated by R848 and then treated with CB one hour later, no reduction in ISRE activation was observed (**Figure 2C**). Further analysis explored CB’s impact on the mRNA levels of several inflammatory cytokines, interferons, and interferon-stimulated genes (ISGs) in PBMCs, assessed via quantitative PCR (q-PCR) (**Figures 2D, E**). Our findings unveil that CB exerts prevention of transcription for key inflammatory markers, including IFN-α, IFN-β, IFN-γ, and several interferon-stimulated genes (ISGs), underscoring its broad immunomodulatory effects. To specifically address CB’s effects on pDCs, we isolated these cells from PBMCs of healthy donors and treated them with CB at 5 and 20 µM concentrations, followed by R848 activation (**Figures 2F, G**). The production of thirteen inflammatory cytokines was measured in the cell culture supernatants using the LEGENDplex assay, revealing that CB significantly prevents the production of various cytokines, notably IFN-α2 and IFN-γ.

### CB reduces IRF7 phosphorylation

**Figure 3:**
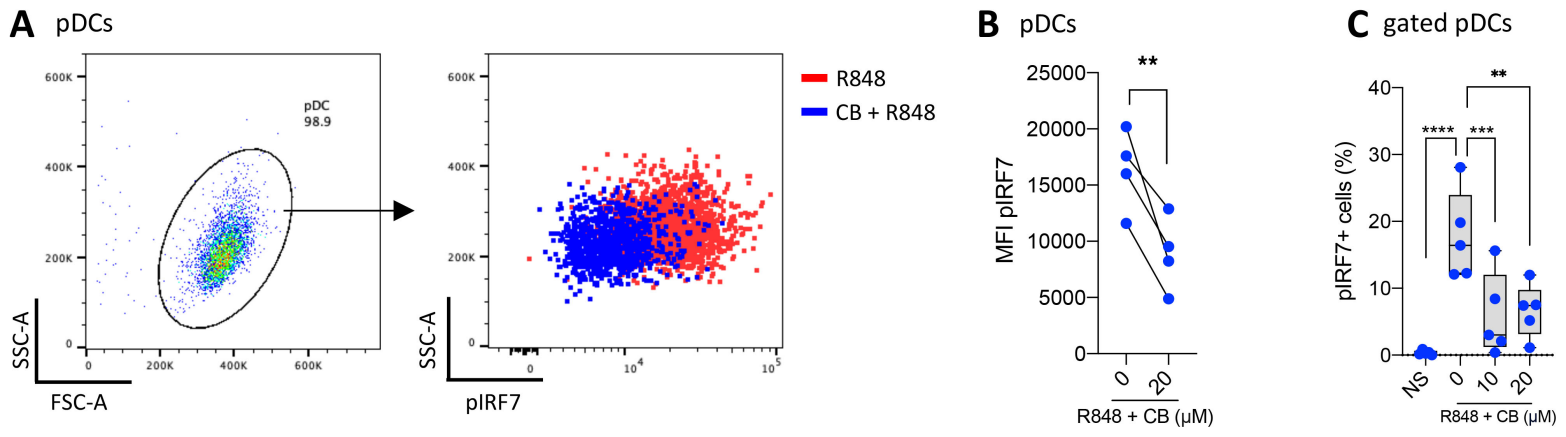
CB prevents IRF7 Phosphorylation in TLR7-Activated pDCs. **(A, B)** pDCs derived from PBMCs of 4 healthy donors were treated with 20 μm CB and then stimulated with R848 for 30 minutes. The levels of pIRF7 were measured using flow cytometry. **(A)** Dot plot representation illustrates the obtained results, and **(B)** pairwise comparison across the 4 donors **(C)** The level of pIRF7 was measured in gated pDCs from whole blood of 5 healthy donors, treated with CB and then stimulated with R848 for 24 hours. Two-way ANOVA with dunnett’s multiple comparisons test. All data are presented as median ± range. ****P < 0.0001, ***P < 0.001, **P < 0.01.

Since the production of IFN-α is mediated by the phosphorylated form of IRF7, we aimed to investigate the state of IRF7 in the presence of CB. pDCs were isolated from PBMCs, treated with CB, and stimulated with R848 for 30 minutes to detect the early induction of pIRF7 (**Figures 3A, B**). CB significantly prevents the early phosphorylation of IRF7 in pDCs. To examine the effect of CB on the later induction of pIRF7, PBMCs were treated with CB and subsequently stimulated with R848 for 24 hours. This experiment demonstrated that CB effectively blocks the late phosphorylation of IRF7 in pDCs isolated from PBMCs (**Figure 3C**).

### CB suppresses IFN production in cells from SLE patients

**Figure 4:**
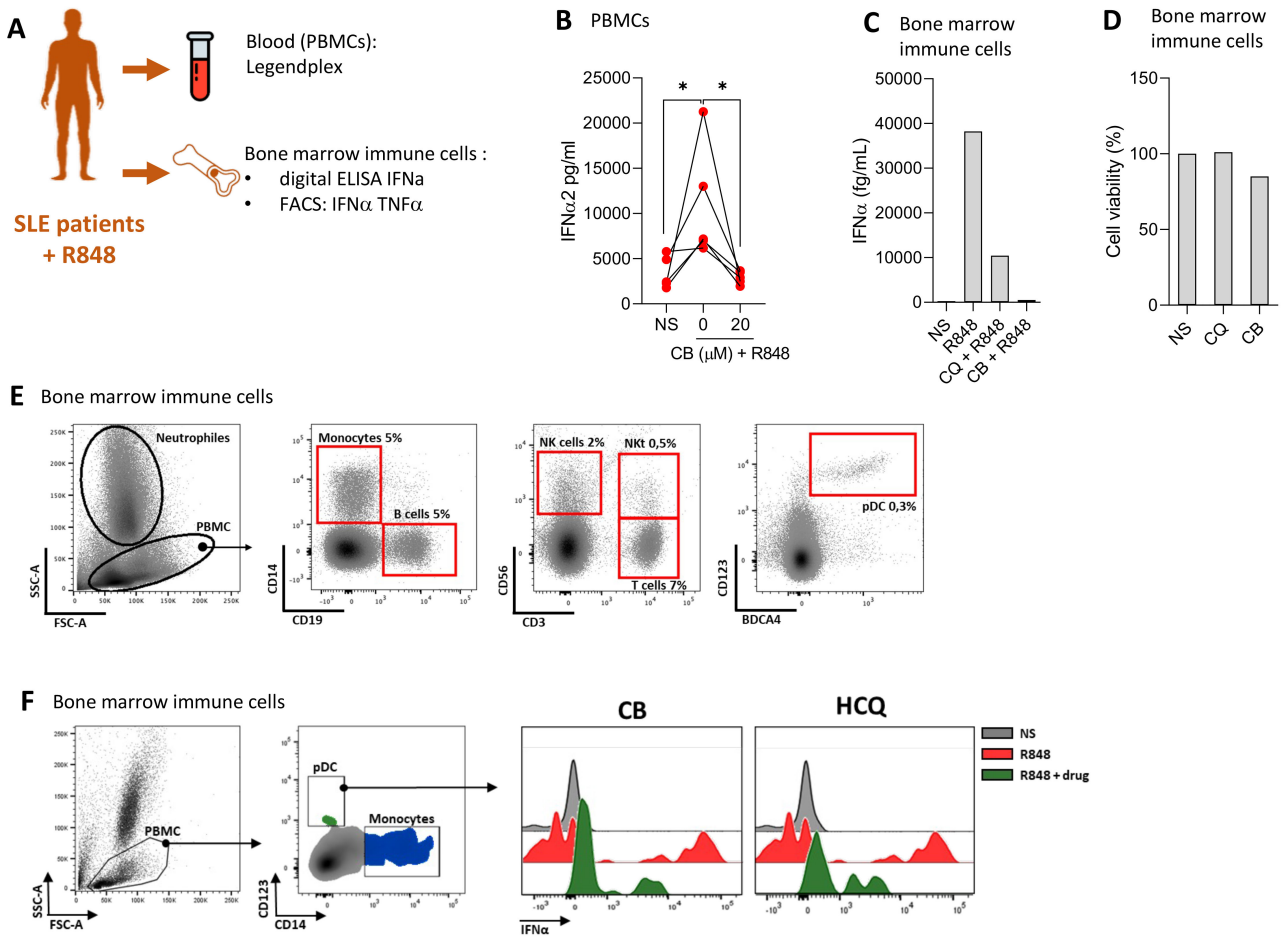
CB controls induced and spontaneous inflammation *in vitro* in cells from patients with SLE. **(A)** Overview of key tests conducted. **(B)** PBMCs from SLE patients were treated with CB (20 μM) and then stimulated with R848. A Legendplex assay was performed on the culture supernatants. Kruskal-Wallis test with Dunnett’s multiple comparisons test. **(C–F)** Effect of CB was evaluated *in vitro* on bone marrow immune cells of one patient with SLE. **(C)** Bone marrow immune cells were cultured with R848 (5 μg/ml) in the presence or absence of CB (20 μM) and chloroquine (20 μM). IFN-α secretion was measured by Simoa. **(D)** Cell viability was measured by Cell-Titer Glo. **(E)** Bone marrow immune cells phenotype of SLE patient was performed by flow cytometry using CD14, CD19, CD56, CD3, CD123 and BDCA4 antibodies. **(F)** Bone marrow immune cells were cultured with R848 (5 μg/ml) in the presence or absence of CB (20 μM) and chloroquine (20 μM). pDCs were identified and IFN-α MFI was quantified by flow cytometry. *P < 0.05. NS, nonstimulated.

We evaluated the ability of CB to modulate immune responses in cells from SLE patients, aiming to assess CB’s efficacy in a disease-relevant context. The experimental setup and key tests conducted are summarized in a schematic (**Figure 4A**). PBMCs from four SLE patients were isolated and then treated with CB and subsequently stimulated with R848. The production of 13 inflammatory cytokines was quantified in culture supernatant using the LEGENDplex assay (**Figure 4B**). This analysis revealed that CB effectively prevents the production of inflammatory cytokines, particularly IFNs, in PBMCs from SLE patients, highlighting its potential as a modulator of disease-associated inflammation. Immune cells from the bone marrow of a patient experiencing an active SLE flare were subjected to CB along with HCQ, the standard SLE treatment. Following R848 stimulation, the level of IFNα was quantified in the culture supernatants using Single Molecule Array (SIMOA) technology (**Figure 4C**). The results demonstrated that CB prevented IFN-α production similarly to HCQ in the bone marrow-derived immune cells of an SLE patient in flare, without inducing toxicity (**Figure 4D**). To further characterize the immune cells isolated from the bone marrow of the SLE patient, phenotyping was performed using flow cytometry (**Figure 4E**). Bone marrow sample contained 0.3% pDCs and within these selected pDCs, cells double-positive for IFNα and TNF-α production were detected under conditions of R848 stimulation, an effect reversed by CB and HCQ treatment (**Figure 4F**).

### Clobenpropit binding mode on CXCR4 and antagonism towards CXCL12

**Figure 5:**
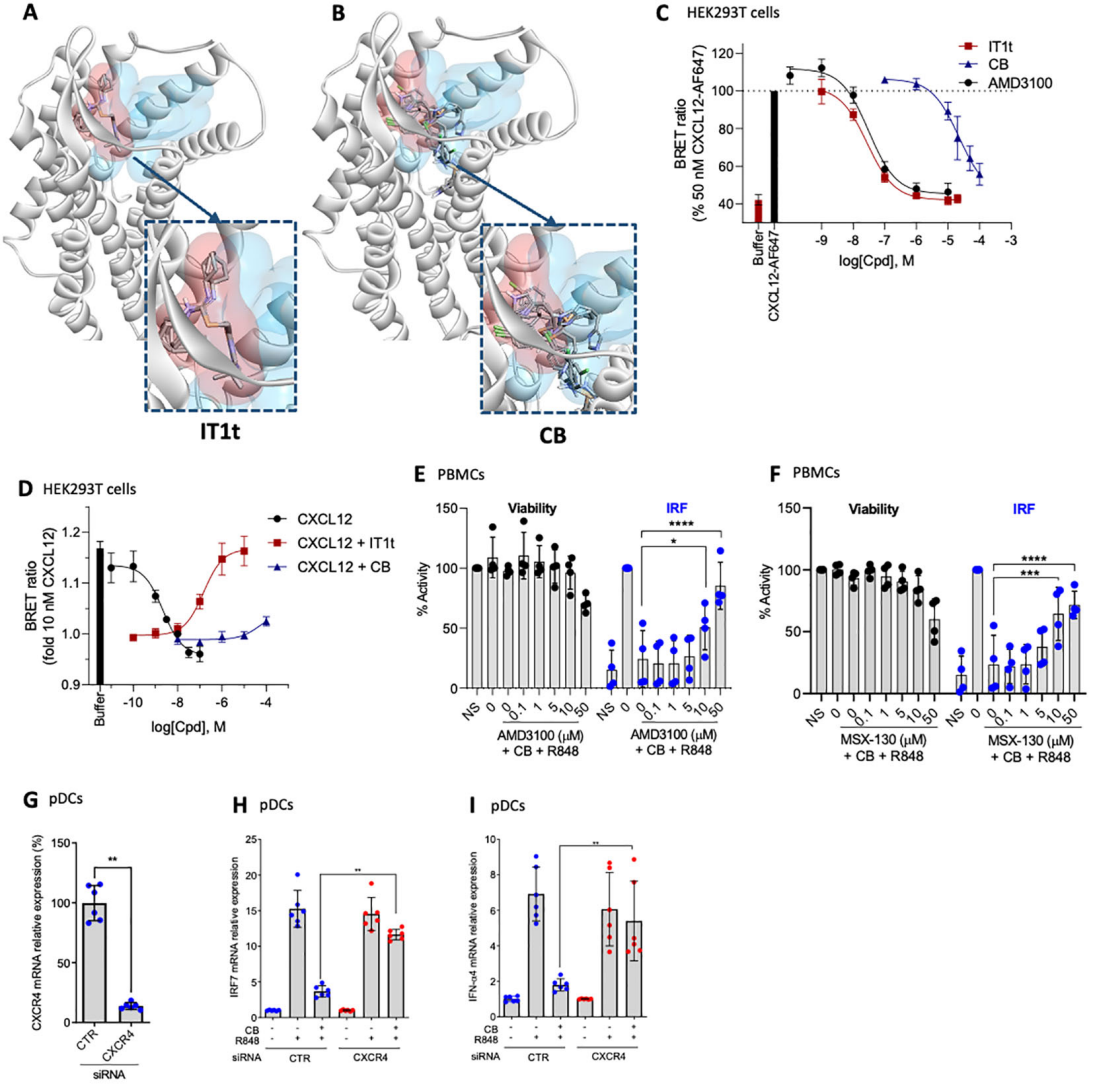
CB controls inflammation in a CXCR4 dependant way. **(A, B)** *In silico* modeling of the CXCR4 receptor, with the minor pocket highlighted in red and the major pocket in blue. Docking of compound **(A)** IT1t and **(B)** CB. **(C)** Change in BRET ratio in HEK293T cells transiently transfected with Nanoluc-CXCR4 at t=24 min upon treatment with 50 nM CXCL12-AF647 and increasing concentrations of unlabelled compound. Data represents means ± S.E.M. of n=3 independent experiments performed in duplicate normalized to 50 nM of CXCL12-AF647. **(D)** Change in BRET ratio in HEK293T cells transiently transfected with CXCR4-WT and Gαi2-NLuc/Gy2-VENUS at t=12 min upon treatment with increasing concentrations of CXCL12 (black) or 10 nM CXCL12 plus increasing concentrations of IT1t (red) or CB (blue). **(E, F)** PBMCs were pre-incubated with a range of **(E)** AMD3100 or **(F)** MSX-130 doses, followed by treatment with 20mM CB and stimulation with R848. Induction of the IRF pathway was assessed using the THP1-Dual reporter cell line from culture supernatants. Cell viability was evaluated using CellTiter Glo. Two-way ANOVA dunnett’s multiple comparisons test. **(G-I)** Human pDCs were sorted from healthy donor PBMCs then treated with siCXCR4 or siScrbl. 20 µM CB were added and cells were then stimulated with 1µg/mL R848. **(G)** siRNA efficiency was measured using PCR. Mann-Whitney test was used. **(H)** IFN-α4, and **(I)** IRF7 mRNA levels were evaluated using PCR. Mann-Whitney test was used. All data are presented as median ± range. ****P < 0.0001, ***P < 0.001, **P < 0.01, *P < 0.05.

The current study further explores the mechanism of action of CB, with particular emphasis on its interaction with CXCR4. Utilizing an *in silico* docking approach, we compared the binding sites of the well described minor pocket ligand IT1t (45) and CB. The analysis revealed distinct binding preferences. IT1t binds into the minor pocket with an overlap with the major pocket targeted by other antagonists such as AMD3100 (plerixafor), an FDA/EMA-approved CXCR4 antagonist (**Figure 5A**). In contrast, CB targets the minor pocket deeper than IT1t, and did not show overlapping with the major pocket (**Figure 5B**), suggesting a unique mode of interaction with the receptor. To further elucidate the functional implications of these binding characteristics, we used a NanoBRET binding assay, using CXCL12-AF647 as a fluorescent probe, to assess the capacity of IT1t and CB to inhibit the binding of the endogenous ligand of CXCR4, CXCL12 (**Figure 5C**). IT1t and AMD3100 effectively inhibit binding of CXCL12-AF647 with pKi values of 7.64 ± 0.12 and 7.59 ± 0.14, respectively. Notably, CB blocks CXCL12-AF647 binding with a 1000-fold lower potency (pKi = 4.73 ± 0.25). However, this indicates that CB very weakly antagonizes CXCL12 while targeting CXCR4. We investigated whether CB has antagonistic activity towards CXCL12 using a G protein activation assay (**Figure 5D**). CXCL12 activates Gαi2 with a pEC_50_ of 8.73 ± 0.29, consistent with previous findings (46, 47). This activation of CXCL12 can be reversed by the simultaneous addition of IT1t with a pIC_50_ of 6.88 ± 0.26. On the other hand, CB appears to have only a small influence on CXCL12 G protein activation, observable primarily at high concentrations (100 µM). To further assess whether the anti-IFN properties of CB are CXCR4 dependent, PBMCs were incubated with the known CXCR4 antagonists AMD3100 (**Figure 5E**) or MSX-130 (**Figure 5F**) prior to the CB/R848 treatment. AMD3100 and MSX-130 negate CB’s anti-IFN effect in a dose-dependent manner. To further investigate whether CB’s anti-IFN activity is CXCR4-dependent and the role of IRF7, the expression of CXCR4 was reduced via the transfection of siRNA in isolated pDCs (**Figure 5G**). On pDCs treated with a control siRNA (CTR), CB prevented the mRNA level encoding for IFN-α (**Figure 5H**) and IRF7 (**Figure 5I**), whereas on pDCs treated with a CXCR4 siRNA, CB loses its ability to modulate the response (**Figures 1I, J**). All these data show that CB directly interacts with CXCR4 and mediates its anti-IFN properties through blocking IRF7 phosphorylation potentially by reducing the amount of total IRF7.

### Therapeutic activity of CB in a pristane induced SLE mouse model

**Figure 6:**
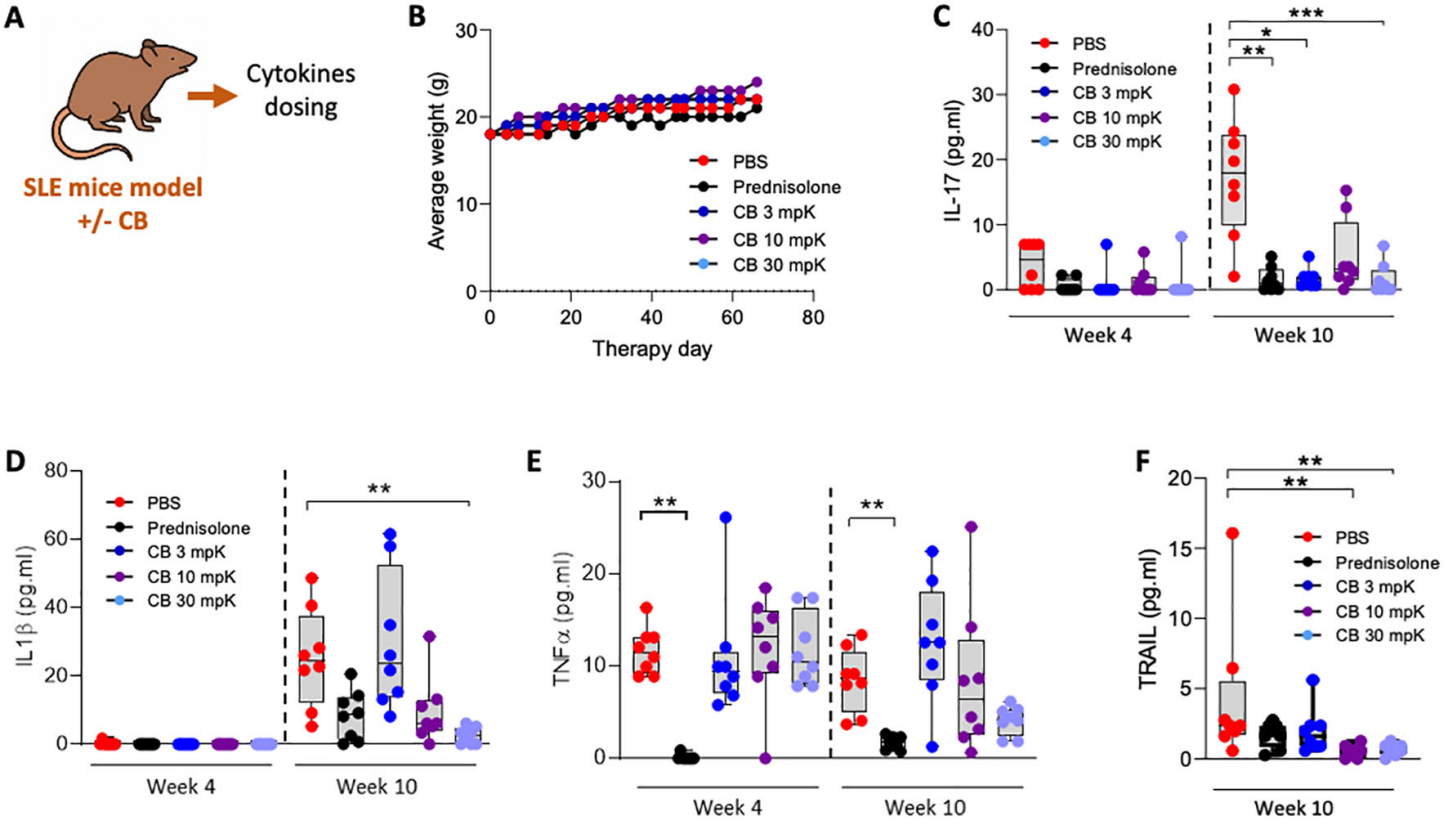
CB controls inflammation and disease onset in SLE mice. **(A)** Overview of key tests conducted. **(B–F)** Mice (n=8/group) were injected intraperitoneally daily with 0.5 ml of pristane to induce disease and treatment consisting of either PBS, prednisolone (15 mg/kg) or 3, 10, or 30 mg/kg of CB for 10 weeks. **(B)** Average body weight of treated mice throughout the experiment. **(C–F)** Inflammatory status was evaluated in the serum of mice by quantifying protein dosage of IL-17 **(C)**, IL-1β **(D)**, TNF-α **(E)**, and TRAIL **(F)**. Kruskal-Wallis test with Dunn’s *post hoc* correction. ***P < 0.001, **P < 0.01, *P < 0.05.

To evaluate CB’s therapeutic potential for SLE, we used the pristane-induced SLE mouse model, which closely replicates the human condition. The experimental setup and key tests conducted are summarized in a schematic (**Figure 6A**). Indeed, pristane-induced SLE aligns closely with the criteria established by the Systemic Lupus Erythematosus International Collaborating Clinics (SLICC) for classifying human lupus. This alignment is evident through the existence of an IFN signature and the presence of anti-dsDNA antibodies (35).

The mice were treated over a 10-week period with PBS (as a placebo), the reference SLE treatment prednisolone at 15 mg/kg, or CB at 3, 10, or 30 mg/kg (n=8 mice per condition). To assess the general condition of the mice, daily weight measurements were recorded throughout the treatment period (**Figure 6B**). Notably, the diverse treatments had no apparent impact on the mice’s weight. Additionally, all treated mice exhibited normal behaviors and showed no signs of distress or illness throughout the study, indicating that the treatments were well tolerated. Key inflammatory cytokines, including IL-17 (**Figure 6C**), IL-1β (**Figure 6D**), TNF-α (**Figure 6E**), and TRAIL (**Figure 6F**), were measured in the blood at both 4- and 10-weeks post-treatment initiation. CB at all tested dosages reduced the levels of these cytokines.

## Discussion

In this study, we demonstrate that histamine and the structural histamine stable analogue CB potently prevents IFN-I production in a variety of TLR7-activated immune cells, particularly in primary pDCs. In addition, CB exhibits broader-spectrum activity in purified pDCs and in a lupus mouse model, significantly reducing levels of pro-inflammatory cytokines IL-1β, IL-17, TNF-α and the interferon-regulated gene TRAIL, both *in vitro* and *in vivo*. Moreover, CB prevents early IRF7 phosphorylation and IRF7 mRNA expression. Reducing CXCR4 expression by siRNA in primary pDCs interfered with CB’s ability to modulate IRF7 transcription and led to the restoration of IFN-I mRNA expression. Finally, we showed that CB displayed minimal antagonistic activity towards CXCL12, a characteristic that might be crucial for avoiding side effects in clinical development.

SLE remains a significant cause of mortality among young women. A meta-analysis of over 26,000 female patients with SLE in the USA revealed a mortality rate 2.6 times higher than the general population. In comparison, the standardized mortality ratio (SMR) exceeded two for cardiovascular disease and approached five for both infection and renal disease (48). The chronic production of type I interferon (IFN-I), a hallmark of SLE (49), impacts both innate and adaptive immunity (50). Indeed, IFN-I induced prolonged survival and activation of B cells, differentiation and class-switch recombination causing enhanced antibody production (51). This leads to the emergence of uncontrolled autoreactive plasmocytes and production of pathogenic autoantibodies (52). *Ex vivo* experiments showed that CB potently prevents IFN-I production by SLE patients’ PBMC. Furthermore, pDC from one patient’s bone marrow activated by R848 produced high levels of IFN-α which was prevented by CB. The potency of CB to reduce intracellular IFN-α in pDC was like the drug HCQ, routinely used in clinics. Nevertheless, our findings on TLR-activated PBMCs demonstrate that CB’s impact wasn’t confined solely to IFN-I but could also encompass all IFN subtypes and pro-inflammatory cytokines.

CB showed interesting *in vivo* anti-inflammatory activity in a pristane-induced SLE by significantly reducing TRAIL, IL-1β and interestingly IL-17. IL-17 is a key cytokine implicated in the pathogenesis of both animal models of autoimmunity and human autoimmune diseases such as SLE (53, 54). Studies showed that patients with SLE exhibit elevated serum levels of IL-17 and an increased number of Th17 cells (55, 56). Additionally, high baseline serum levels of IL-17 have been linked to poor histopathological outcomes following immunosuppressive therapy (57). CB activity is thus beyond type I IFN by reducing inflammation. This *in vivo* anti-inflammatory activity was similar to the referenced corticosteroid prednisolone. In contrast, CB poorly reduces the levels of anti-dsDNA antibodies in SLE mice (data not shown) compared to prednisolone.

While the central role of IFN-I in SLE pathogenesis is undisputed, recent studies have highlighted the crucial involvement of type III IFN and inflammation in the progression of the disease (58, 59). Although, clinical trials showed that targeting only IFN-I in SLE may not be sufficient (60, 61). We showed that mRNA of IFN-III and protein was statistically reduced in TLR-activated PBMC by CB exposure. Several studies revealed elevated serum concentrations of type III IFNs in SLE patients compared to healthy controls, with even higher levels observed in those with active SLE. These type III IFN levels were found to be correlated with SLE Disease Activity Index scores and titers of anti-double-stranded DNA (dsDNA) autoantibodies. Recently, a role for IFN-λ in murine SLE was reported in a TLR7-induced lupus mouse model (62). When Ifnlr1-deficient mice were exposed to the TLR7 agonist imiquimod, they exhibited reduced myeloid expansion and T cell activation, along with decreased skin and kidney inflammation and less immune complex deposition. Imiquimod’s chronic application induces skin inflammatory disease, leading to systemic autoimmunity likely due to epidermal barrier disruption. Our findings indicate that CB exhibited a preventive activity on both IFN-I and IFN-III therefore placing CB and its derivatives as a particularly promising therapeutic strategy.

Accumulating evidence implies that IRF7 is a susceptibility locus for SLE (22). IRF7 mRNA expression is significantly increased in SLE patients, and genetic polymorphisms near/in IRF7 have been substantiated to be related to the onset of SLE (24). SLE patients treated with autologous stem cell transplantation show that high expression of IRF7 is correlated with recurrent lupus disease activity (63). Thus, regulating IRF7 involved in TLR7 and 9 signaling cascade may represent a key avenue for novel therapeutic approaches. Here, we demonstrate that the significant increase in IRF7 phosphorylation induced after 30 minutes of R848 treatment is statistically suppressed by CB. This indicates that CB shuts down TLR7 signaling in the early stages. We further show that this reduction of IRF7 phosphorylation is maintained over time after 24 hours in whole blood stimulated by R848. We next show that CB downregulate IRF7 and IFN-α mRNAs in primary pDC activated by R848. This reduction of IRF7 mRNA expression by CB was abrogated using CXCR4 siRNA in pDC. In contrast to CB, AMD3100 did not modify the level of IRF7 phosphorylation in pDC (data not shown). The distinct ability of CB to regulate IRF7 expression highlights how the minor pocket of CXCR4, in contrast to the major pocket targeted by AMD3100, influences the TLR7 pathway by inhibiting key signaling steps, *ie* IRF7 phosphorylation. Further experiments will be needed to fully decipher the intracellular pathway activated by CB leading to reduction of IRF7 phosphorylation.

The GPCR super family, of which CXCR4 is a member, is the major family of receptors targeted by therapeutic drugs with roughly 1/3 of newly FDA approved drugs (64, 65). This underscores the pivotal role of GPCRs in modern pharmacology and highlights the therapeutic potential of CXCR4. Thus, a more comprehensive analysis of CXCR4 non-canonical signaling pathways will participate in a wider understanding of the implication of GPCRs in immunology and their crosstalk with immune sensors. Dysregulation of the CXCR4/CXCL12 axis is associated in multiple diseases including type I interferonopathies (30). CXCR4 expression is significantly upregulated in SLE patients’ B cells (66), monocytes and neutrophils, and positively correlates with disease progression (30, 66–68). We have previously shown that women exhibited significantly higher baseline levels of CXCR4 as compared to men, and gender accounted for 10% of the naturally observed variability in this gene (69). To put this in context, CXCR4 was in the top 10 most differential genes between men and women in this study. Given that women are at significantly greater risk of developing autoimmunity diseases (70), targeting the CXCR4 anti-inflammatory pathway may therefore represent a particularly effective strategy for these pathologies. However, many clinical trials have been halted due to severe side effects due to CXCL12 antagonism and the main CXCR4 antagonist used in the clinic is AMD3100 to boost stem cell mobilization in lymphoma and myeloma patients (71). These highlight the need for new CXCR4 ligands with diverse signaling patterns and novel modes of action that do not turn off CXCR4/CXCL12 signaling entirely, but selectively modulate downstream pathways. Over the two last decades, several studies identified non-canonical ligands for CXCR4, such as macrophage inhibitory factor (MIF), β-defensin, HIV protein gp120 and extracellular ubiquitin (72). We demonstrate here that CB’s anti-IFN-I activity is mediated through its interaction with CXCR4, with very low antagonism to CXCL12. The *in silico* analysis revealed that the CB binding site was deep into CXCR4’s minor pocket and did not overlap with the major pocket. In contrast, the binding site of IT1t overlaps with the major binding pocket (antagonist) of CXCR4 (45) thus providing an antagonist activity towards CXCL12 as observed in the G protein activation assay and in agreement with already published data (73). This specific binding region deep into CXCR4’s minor pocket provides a unique property to CB with anti-IFN activity without disrupting CXCL12 signaling. This new type of ligands could serve as a novel approach for developing therapeutic molecules targeting CXCR4, potentially bypassing the clinical side effects associated with CXCL12 antagonism. This binding characteristic of CB underscores the therapeutic promise of targeting CXCR4’s minor pocket for autoimmune and inflammatory diseases. The next steps will involve characterizing pathways activated by ligands of the CXCR4 minor pocket in detail to understand the crosstalk between CXCR4 and TLR7 pathways. We initiated a study of the involvement of G proteins or β-arrestin in the pathway induced by minor pocket ligands including all signaling pathways which have been published to exclusively be activated by the minor pocket ligand IT1t (28, 29). Initial experiments using pertussis toxin to inhibit all Gi signaling did not alter CB’s anti-inflammatory activity, indicating the activation of a non-canonical signaling pathway (data not shown). We will persist in our investigation and meticulously delineate the mechanism through which CB activates CXCR4, ultimately resulting in the reduction of IRF7 phosphorylation.

In summary, our findings demonstrate that the structural histamine analogue CB induce a non-canonical activation of CXCR4 that prevents IFN-I production with very weak antagonism towards CXCL12 binding, preventing potential therapeutical side effects. The mechanism of action involves reduction of IRF7 phosphorylation, an early event in TLR7 activation. Furthermore, the pathway induced by CB not only prevents IFN-I but also reduces pro-inflammatory cytokine levels *in vitro*, *ex vivo* and *in vivo*, as evidenced in a pristane-induced lupus mouse model. Consequently, our study proposes a new histamine-based drug strategy targeting the minor pocket of CXCR4 without antagonist activity. This approach, which simultaneously inhibits multiple factors of SLE pathophysiology, including immune cell activation, protein phosphorylation, and inflammatory cytokine production, could represent a promising therapeutic solution. Furthermore, this strategy has the potential to extend its benefits to other autoimmune and inflammatory disorders, opening new paths in their treatment.

## Materials and methods

### *In silico* modeling of the CXCR4 receptor

Docking was performed using FlexX in LeadIT Version: 2.3.2 using default settings. The extended binding pocket in receptor 3ODU was defined around ligand IT1t by selecting residues within a distance of 16A. Ligand preparation was done using Datawarrior 5.5.0. Docking was performed with 1000 maximum number of solutions per iteration and 200 maximum number of solutions per fragmentation. The 10 best scored poses were kept after docking. Protein overlay and poses visualization was performed using Discovery Studio Visualizer v21.1.0.20298.

### Blood samples isolation and culture of blood leukocytes

Blood samples were collected from healthy donors through the French Blood Establishment (“Etablissement Français du Sang”) under the authorization number 07/CABANEL/106, in Paris, France. The procurement of materials from SLE patients was approved by the French Ethical Committee (“Comité de Protection des Personnes”), with the approval number EudraCT: 2018-A01358-47. All experimental protocols involving human blood were in strict compliance with the European Union regulations and the Helsinki Declaration. Consent was duly obtained from every participant, encompassing healthy subjects as well as SLE patients. A summary of the clinical information of the SLE patients is presented in **Table 1**. The *in vitro* studies utilized human mononuclear cells from peripheral blood or synovial fluid, isolated through centrifugation over a density gradient medium (STEMCELL Technologies). Isolation of human pDCs was achieved by negative selection with the EasySep Human Plasmacytoid DC enrichment kit (STEMCELL Technologies). Peripheral blood mononuclear cells (PBMC), and pDCs were incubated in RPMI 1640 medium (Invitrogen, Gaithersburg, MD), enriched with 10% heat-inactivated fetal bovine serum and 1mM glutamine (Hyclone, Logan, UT).

**Table 1:** Patient information.

Patient	Gender	Age	Disease	SLEDAI	Treatment
P1	F	38	SLE	2	prednisone, hydroxychloquine, MMF
P2	F	39	SLE	4	prednisone, nivaquine
P3	F	21	SLE	10	prednisone, hydroxychloquine, MMF
P4	F	33	SLE	2	hydroxychloquine
P5	F	34	SLE	0	prednisone, hydroxychloquine, MMF
P6	F	26	SLE	6	hydroxychloquine, MMF

MMF, mycophenolate mofetil. SLEDAI, Systemic Lupus Erythematosus Disease Activity Index, a clinical tool quantifying SLE activity based on symptoms and lab findings; higher scores indicate more severe disease.

### TMCs isolation from tonsils

The procurement of tonsils received approval from the Ethical Committee (“Comité de Protection des Personnes”), with the identification number ID-RCB/EUDRACT: 2018-A01358-47. Tonsils removed from children diagnosed with obstructive sleep apnea during partial tonsillectomy procedures were processed. These tissues were preserved in 1× phosphate-buffered saline (PBS) for a duration not exceeding two hours, subsequently diced into smaller fragments, and mechanically disrupted with a glass pestle against a 60-mesh steel grid. The resultant Tonsil Mononuclear Cells (TMCs) were then strained through a 70-μm filter and rinsed with PBS. A Ficoll gradient centrifugation was employed to eliminate remaining epithelial cells and debris. The isolated TMCs were maintained in R10 medium. The sample donors were cataloged sequentially from numbers 1 to 7.

### Cell stimulation

PBMCs were initially plated at a density of 2×10^6^ cells per milliliter, and pDCs derived from PBMCs were plated at 1×10^6^ cells per milliliter. Before undergoing a 16-hour stimulation with R848 at a dose of 5 µg/mL or another indicated concentration, cells received a 1-hour pretreatment with AMD3100 (Sigma-Aldrich) or MSX-130 (MedChemExpress), and/or CB (Sigma-Aldrich) at 20 µM or another specified concentration. Following this, cells were analyzed using flow cytometry, or supernatants were collected for cytokine detection. Brefeldin A (BFA) was introduced to the cultures 30 minutes post-stimulation and maintained for 5 hours to facilitate intracellular staining.

### Flow cytometry

Cells were first washed with PBS and then treated with Zombie Aqua viability dye (Biolegend) for 30 minutes at 37°C. After another wash, they were resuspended in PBS containing 2% FCS and 2mM EDTA. For surface marker identification, cells were stained with anti-CD14, anti-CD19, anti-CD56, anti-CD3, anti-CD14 anti-CD123 and anti-BDCA4 antibodies (clone REA) from Miltenyi Biotec at a dilution of 1:100. This involved fixing the cells with 250 µL of for 20 minutes at room temperature, followed by washing and staining with 100 µL of Inside Perm solution (Miltenyi) mixed with anti-IFN-α, anti-TNF-α and/or anti-pIRF7 antibodies at a dilution of 1:50 for 30 minutes at room temperature. Data acquisition was performed on a Canto II flow cytometer and analyzed with Diva software (BD Biosciences, San Jose, CA) and FlowJo software (Treestar, Ashland, OR).

### CXCR4 knock-down experiments

In the CXCR4 knock-down study, pDCs were plated at a concentration of 10^5^ cells per 100 µl in 96-well plates and incubated at 37°C. Two types of siRNA were prepared: control siRNA (Qiagen) and CXCR4-specific siRNA (SMARTPool, Dharmacon), each diluted in DOTAP (1,2-dioleoyl-3-trimethylammonium-propane; Roche Applied Sciences). This mixture was gently stirred and left to incubate at room temperature for 15 minutes. After this incubation, the mixture was introduced to the cells to reach a final siRNA concentration of 160 nM. Following this addition, the cells were incubated for an additional 24 hours at 37°C before proceeding with further treatment and stimulation protocols.

### Real-time quantitative RT-PCR

Cellular RNAs were extracted using the RNeasy Mini kit (Qiagen) following the manufacturer′s instructions. RNA concentration and purity were evaluated by spectrophotometry (NanoDrop 2000c, Thermo Fisher Scientific). A maximum of 500 ng of RNA were reverse transcribed with both oligo dT and random primers using a PrimeScript RT Reagent Kit (Perfect Real Time, Takara Bio Inc.) in a 10 µL reaction. Real-time PCR reactions were performed in duplicate using Takyon ROX SYBR MasterMix blue dTTP (Eurogentec) on an Applied Biosystems QuantStudio 5 (Thermo Fisher Scientific). Transcripts were quantified with the following program: 3 min at 95°C followed by 40 cycles of 15 s at 95°C, 20 s at 60°C and 20 s at 72°C.

Primers used for quantification of transcripts by real time quantitative PCR are the following:

RPL13A: forward primer, 5’- AACAGCTCATGAGGCTACGG-3’; reverse
primer, 5’- TGGGTCTTGAGGACCTCTGT-3’
CXCR4: forward primer, 5’-GCATGACGGACAAGTACAGGCT-3’; reverse
primer, 5’-AAAGTACCAGTTTGCCACGGC-3’
IRF7: forward primer, 5’-TACCTGTCACCCTCCCCAAG-3’; reverse
primer, 5’-CGGCCCTTGTACATGATGGT-3’
IFN-α2: forward primer, 5’-CTTGACTTGCAGCTGAGCAC-3’; reverse
primer, 5’-GCTCACCCATTTCAACCAGT-3’
IFN-α4: forward primer, 5’-CCCACAGCCTGGGTAATAGGA-3’; reverse
primer, 5’-CAGCAGATGAGTCCTCTGTGC-3’
IFN-β: forward primer, 5’-TGCTCTCCTGTTGTGCTTCTC-3’; reverse
primer, 5’-CAAGCCTCCCATTCAATTGCC-3’
IFN-γ: forward primer, 5’-GGCAGCCAACCTAAGCAAGAT-3’; reverse
primer, 5’-CAGGGTCACCTGACACATTCA-3’
IFN-λ1: forward primer, 5’-TTCCAAGCCCACCACAACTG-3’; reverse
primer, 5’-GTGACTCTTCCAAGGCGTCC-3’
CCL5: forward primer, 5’-CTGCTTTGCCTACATTGCCC-3’; reverse
primer, 5’-TCGGGTGACAAAGACGACTG-3’
CXCL10: forward primer, 5’-CGCTGTACCTGCATCAGCAT-3’; reverse
primer, 5’-GCAATGATCTCAACACGTGGAC-3’
CXCL12: forward primer, 5’-AGATGCCCATGCCGATTCTT-3’; reverse
primer, 5’-AGGGCACAGTTTGGAGTGTT-3’
Mx1: forward primer, 5’-AAGCTGATCCGCCTCCACTT-3’; reverse
primer, 5’-TGCAATGCACCCCTGTATACC-3’
Mx2: forward primer, 5’-GAAAAGCGTCATGAATGTGGT-3’; reverse
primer, 5’-TCAGCCTGTTTGTGATCTCCT-3’
IFIT1: forward primer, 5’-ATGCGATCTCTGCCTATCGC-3’; reverse
primer, 5’-CCTGCCTTAGGGGAAGCAAA-3’
ISG15: forward primer, 5’-CAGCGAACTCATCTTTGCCAG-3’; reverse
primer, 5’-GACACCTGGAATTCGTTGCC-3’
ISG20: forward primer, 5’-TGACCTGAAGCACGACTTCC-3’; reverse
primer, 5’-CACAACAGCCTGTCAGTGGA-3’
IFI6: forward primer, 5’-GGGTGGAGGCAGGTAAGAAA-3’; reverse
primer, 5’-GTCAGGGCCTTCCAGAACC-3’
IFI27: forward primer, 5’-ATCAGCAGTGACCAGTGTGG-3’; reverse
primer, 5’-GGCCACAACTCCTCCAATCA-3’
ISG54: forward primer, 5’-AGAGCGAAGGTGTGCTTTGA-3’; reverse
primer, 5’-CTGAGATGGTGGCCAGTTGT-3’
ISG56: forward primer, 5’-AGGACAGGAAGCTGAAGGAG-3’; reverse
primer, 5’-AGTGGGTGTTTCCTGCAAGG-3’
IFITM1: forward primer, 5’-AGGAAGATGGTTGGCGACG-3’; reverse
primer, 5’-GCCGAATACCAGTAACAGGATGA-3’
IFITM2: forward primer, 5’-TTGTGCAAACCTTCTCTCCTGT-3’; reverse
primer, 5’-CCCAGCATAGCCACTTCCTG-3’
IFITM3: forward primer, 5’-GAAGATGGTTGGCGACGTGA-3’; reverse
primer, 5’-CACTGGGATGACGATGAGCA-3’
TRAIL: forward primer, 5’-GCTGAAGCAGATGCAGGACAA-3’; reverse
primer, 5’-TGACGGAGTTGCCACTTGACT-3’
PML: forward primer, 5’-ATCACCCAGGGGAAAGATGC-3’; reverse
primer, 5’-TGAACCTGGGCCTTCACTCT-3’
TRIM22: forward primer, 5’-GCCCTGCAGAGGCTGATAAA-3’; reverse
primer, 5’-TGGATATAATTCTTCCAGGCGGT-3’
OAS1: forward primer, 5’-GCTGAGGCCTGGCTGAATTA-3’; reverse
primer, 5’-CAGTCCTCTTCTGCCTGTGG-3’
RIG-I: forward primer, 5’-ATCCAAACCAGAGGCAGAGGAA-3’; reverse
primer, 5’-ACTGCTTCGTCCCATGTCTGAA-3’
BST-2: forward primer, 5’-TCATCGTGATTCTGGGGGTG-3’; reverse
primer, 5’-GTTGCAGGAGATGGGTGACA-3’
RSAD2: forward primer, 5’-TTGAGTGTGTTCAGGCAACCT-3’; reverse
primer, 5’-TTGGTAGCTAGCAGCCAGAAG-3’
Zap: forward primer, 5’-CCAACATTTGTGCCTCAGTGG-3’; reverse
primer, 5’-ACTGACGAGGTCTTTGCTGG-3’
TRIM5: forward primer, 5’-ACTGAGATGGTGCAGCAGAC-3’; reverse
primer, 5’-GGTCACGTTCTCCGTCCTTT-3’

### Cytokine detection

To assess cytokine production, supernatants were analyzed using the LEGENDplex Antivirus Human Panel bead assay (Biolegend, San Diego, USA), executed in accordance with the instructions provided by the manufacturer.

To quantify the release of active IFN from TMCs, we employed a biological assay involving a stable cell line (STING37) in which a luciferase reporter gene is controlled by five IFN-stimulated response elements. Initially, supernatants from TMCs were collected following a 24-hour stimulation and stored at -20°C. These TMCs supernatants were subsequently distributed into the culture wells of a 96-well plate, with each well containing 35,000 STING37 cells. After an additional 24-hour incubation, luciferase activity was assessed by introducing 50 μl of Bright-Glo reagents (Promega) to the culture wells and measuring bioluminescence using a luminometer.

### Simoa

A Simoa digital ELISA designed for the specific detection of IFN-α2 was established using the Quanterix Homebrew Assay (74). The BMS216C (eBioscience) antibody clone was used as a capture antibody after coating on paramagnetic beads (0.3 mg/mL), and the BMS216BK already biotinylated antibody clone was used as the detector at a concentration of 0.3ug/mL. The SBG revelation enzyme concentration was 150 pM. Recombinant IFN-α2c was used as calibrator. The limit of detection was determined by calculating the mean value of all blank runs plus three standard deviations (SDs), resulting in a limit of detection of 0.23 fg/mL.

### Compound binding

NanoLuc binding assays were slightly modified from (White et al.) (75). In brief, HEK293T cells were transiently transfected with 100 ng/well Nanoluc-CXCR4 and seeded at 10,000 cells/well into poly-D-lysine coated, white flat bottom 96-well plates 48h before the experiments and incubated at 37°C/5% CO_2_. On the day of the experiment, cells were washed once with HBSS + 0.1% BSA before unlabelled compounds or buffer and 10 µM furimazine were added for 5 min and read twice on a Varioskan Lux using a 460 nm (80 nm bandpass) and a >610 nm (longpass) filter. Subsequently CXCL12-AF647 was added, and luminescence was monitored in the same manner for further 36 min every 3 min. The BRET ratio was calculated by dividing the 610 nm emission by the 460 nm emission.

### G protein activation

G protein activation assays are performed as described in (Schihada et al., 2021) (76). HEK293T cells were transiently transfected with 50 ng/well CXCR4 WT and 50 ng/well G_i2_ construct and seeded at 30,000 cells/well into poly-D-lysine coated, white flat bottom 96-well plates 48h before the experiments and incubated at 37°C/5% CO_2_. On the day of the experiment, cells were washed once with HBSS + 0.1% BSA buffer before IT1t or CB and 10 µM furimazine were added for 5 min and the plate was read three times on a Varioskan Lux using a 460 nm (80 nm bandpass) and a 530 nm (30 nm bandpass) filter. Subsequently CXCL12 was added, and luminescence was monitored in the same manner for further 30 min every 3 min. The BRET ratio was calculated by dividing the 530 nm emission by the 460 nm emission.

### *In vivo* treatment of mice

Animal experiments were conducted under blind conditions by Washington Biotechnology, Inc. The animals were housed in a controlled environment with regulated temperature and humidity, following a 12-hour light/dark cycle, and had unrestricted access to food and water. The Animal Care and Use Committee at Washington Biotechnology, Inc. reviewed and approved all mouse experiments (IACUC no. 17-006). Animal welfare and experimental protocols strictly adhered to the principles outlined in the Guide for the Care and Use of Laboratory Animals and complied with ethical guidelines and regulations.

For the pristane injection procedure, each mouse in all experimental groups received an intraperitoneal injection of 0.5 ml of pristane (catalog no. P9622, Sigma-Aldrich, BioReagent). This was used to induce a systemic lupus erythematosus (SLE) model in female BALB/c mice (20-25g, ENVIGO, R# 3859), aged 8-10 weeks. The mice were quarantined, tagged, and monitored to ensure good health before the start of the study. The choice of female mice reflects the higher prevalence of SLE in females, making the model more representative of human disease.

Treatment with test compounds, including CB, began on the same day as the pristane injection, with the first dose administered one hour after the injection. Prednisolone (4.5 mg; catalog no. P4153, Sigma-Aldrich) was used as a positive control and was dissolved in 3 ml of PBS to create a solution with a concentration of 1.5 mg/ml. In group 2, all mice were administered daily oral gavage of prednisolone at a dose of 15 mg/kg. CB was dissolved in PBS and administered via intraperitoneal injection at daily doses of 3 mg/kg, 10 mg/kg, or 30 mg/kg, in groups 3 to 5.

Body weights and general health conditions were monitored throughout the 10-week study to assess any potential toxicity, with body weight loss kept under 20%. Blood samples were collected via the orbital sinus at multiple time points (weeks 4, 8, and 10) and processed to serum for analysis. Anti-dsDNA antibodies were measured as a primary readout, along with cytokine levels (TNF-α, IL-1β, IL-17, and TRAIL) by ELISA, to evaluate the efficacy of CB. Spleen and kidney samples were collected at the end of the study for further analysis.

For ELISA assays, the following kits were used in accordance with the manufacturer’s instructions: TNF-α ELISA kit (catalog no. MTA00B, R&D Systems), IL-1β ELISA kit (catalog no. MLB00C, R&D Systems), IL-17 ELISA kit (catalog no. M1700, R&D Systems), and TRAIL ELISA kit (catalog no. ELM-TRAIL-1, RayBiotech).

mg/kgmg/kgmg/kg

## Data Availability

The original contributions presented in the study are included in the article/supplementary material. Further inquiries can be directed to the corresponding authors.
